# Histological grading of breast cancer on needle core biopsy: the role of immunohistochemical assessment of proliferation

**DOI:** 10.1111/j.1365-2559.2010.03620.x

**Published:** 2010-08

**Authors:** T’ng Chang Kwok, Emad A Rakha, Andrew H S Lee, Matthew Grainge, Andrew R Green, Ian O Ellis, Desmond G Powe

**Affiliations:** Department of Pathology and School Molecular Medical Sciences, Nottingham University HospitalNottingham, UK; 1 Division of Epidemiology and Public Health, School of Community Health Sciences, University of Nottingham Medical SchoolNottingham, UK

**Keywords:** breast neoplasm, histological grading, needle core biopsy

## Abstract

**Aims:**

Histological grade assessed on needle core biopsy (NCB) moderately concurs with the grade in the surgical excision specimen (SES) (κ-values between 0.35 and 0.65). A major cause of the discrepancy is underestimation of mitoses in the NCB specimen. The aim was to determine the best method of assessing proliferation on NCB.

**Methods and results:**

Proliferative activity of 101 invasive carcinomas of the breast on NCB and SES was assessed using mitotic counts on routine haematoxylin and eosin (H&E) sections and immunohistochemical markers Mib-1 and phosphorylated histone H3 (PPH3). H&E mitotic count in SES was considered as the gold standard. H&E mitotic count was found to be underestimated on NCB when compared with that in SES (*P* < 0.001), but no significant difference was detected between NCB and SES regarding Mib-1 (*P* = 0.13) or PPH3 (*P* = 0.073). Using receiver–operating characteristic curve, Mib-1 on NCB was found to agree with the gold standard significantly better than routine H&E on NCB.

**Conclusions:**

Immunohistochemical markers in NCB showed better concordance with H&E mitotic count in SES (gold standard) than routine H&E mitotic count in NCB. Further refinement of cut-offs and scoring methods is needed.

## Introduction

Needle core biopsy (NCB) is routinely used for the preoperative diagnosis of breast lesions. It can also provide prognostic and predictive information.^1^ NCB may be the only pretreatment sample for patients showing complete pathological response to neoadjuvant therapy,^2^,^3^ which is increasingly used to reduce micrometastatic disease and down-stage primary tumours, allowing conservative surgery to be performed.^4^

Histological grade is a powerful independent prognostic factor based on assessment of tubule formation, nuclear pleomorphism, and mitotic count.^5^ Histological grade assessed in NCB moderately concurs with the grade in the surgical excision specimen (SES) (κ-values between 0.35 and 0.65).^2^,^6^–^13^ Core biopsy grade tends to be lower, particularly due to underestimation of mitotic count.^7^–^12^ This may potentially exclude patients that would benefit from neoadjuvant therapy.^14^

Moreover, cell proliferation determines clinical outcome in breast cancer,^15^ predicting response to chemotherapy.^16^,^17^ Most tumour microarray gene expression studies have found cell cycle regulation genes to be highly expressed in breast cancer with poor outcome.^18^ These emphasize the importance of proliferation assessment in breast cancer, not only on SES but also on NCB.

Thus, there is a need for a rapid, objective, reproducible and perhaps automated NCB proliferation marker. Several candidates have been proposed.

Ki67, a cell proliferation-related human nuclear antigen,^19^ is expressed in cycling cells from G_1_ to M phase, but not in quiescent G_0_ phase. Monoclonal antibody Mib-1^19^ targets recombinant fragments of the Ki67 antigen gene, allowing growth fraction to be determined in formalin-fixed paraffin-embedded (FFPE) specimens. Some report that Mib-1 growth fraction is superior to routine haematoxylin and eosin (H&E)-based mitotic counting, the histopathology gold standard.^20^ However, others argue that Mib-1 immunopositive cells may undergo apoptosis before entering mitosis.^21^,^22^

Another proliferation biomarker is histone H3. The phosphorylation of histone H3 occurs exclusively during mitosis, and is required for initiating and coordinating chromosome condensation and decondensation.^23^,^24^ H&E mitotic counting correlates well with mitotic counting using anti-phosphorylated histone H3 (PPH3) immunohistochemistry.^25^ Some suggest PPH3 detection is prognostically superior to H&E,^26^,^27^ but insufficient studies exist to achieve consensus.

This study assessed the proliferative activity of breast cancer on NCB using H&E, and immunohistochemistry for Mib-1 and PPH3. The results were compared with H&E mitotic count in SES as the proliferation assessment gold standard to determine the accuracy of these proliferation markers on NCB. Our aims were to explore methods of improving breast cancer proliferation assessment on NCB.

## Materials and methods

### Patient selection

One hundred and fifty-five cases were reviewed (A.H.S.L.) from consecutive patients attending the Breast Unit at Nottingham City Hospital. Included in this study were 101 consecutive patients who satisfied the following criteria: at least 10 high-power fields (HPF) of invasive carcinoma in both NCB and SES (×400, field diameter 0.61 mm) and <2 months’ duration between NCB and the SES procedure to exclude any patients receiving neoadjuvant systemic therapy.

On average, two core biopsy specimens (range 1–5 cores) were taken using a 14-G needle automated core biopsy gun, except for one patient who was biopsied using a mammotome. Eighty-nine NCBs were ultrasound guided, three used stereotactic instrumentation and three were obtained freehand, while no information was available for the remaining six NCBs.

The median patient age was 61 years (range 35–88). The median tumour size was 15 mm (range 2.5–60). The tumours were of the following histological types: no special type (*n* = 61), lobular (*n* = 11), tubular (*n* = 3), mucinous (*n* = 2), medullary-like (*n* = 2), cribriform (*n* = 1) and mixed (*n* = 21). This study was approved by the Nottingham Research Ethics Committee.

All the NCB specimens and SES were managed according to the UK National Health Service Breast Screening Programme 2005 guidelines.^28^ NCB specimens were fixed directly in formalin for at least 8 h, while in the SES a cruciate incision was made on the posterior aspect of the tumour immediately on arrival in the department before fixation to ensure adequate fixation of tumour tissue. The SES was then fixed in sufficient volume of formalin for 48 h. Samples were routinely processed and embedded in paraffin wax (FFPE).

### Mitotic counts

Mitotic counts were assessed in 2 μm H&E-stained FFPE sections. The highest count in 10 HPF (×400, field diameter 0.61 mm) was recorded in both NCB and SES, as part of the routine reporting, by experienced breast pathologists. The mitotic counts were divided into three mitotic scores: mitotic score 1 for 0–10 mitoses/10 HPF, mitotic score 2 for 11–21 mitoses/10 HPF and mitotic score 3 for ≥22 mitoses/10 HPF,

### Immunohistochemistry

Tissue sections (4 μm) were dewaxed in xylene and rehydrated before microwave antigen retrieval in 0.01 m sodium citrate buffer at pH 6. Sections were stained using Mib-1 (DakoCytomation, Carpinteria, CA, USA; M7240) and PPH3 (Upstate Biotechnology, Billerica, MA, USA; 06-570) antibodies. For both SES and NCB 1:50 Mib-1 dilution was used; 1:400 PPH3 dilution was used for SES while 1:500 PPH3 dilution was used for NCB due to denser PPH3 immunoreactivity in NCB. A labelled streptavidin–biotin technique on a Dako Techmate™ 500 Plus automated immunostainer (Dako Ltd, Ely, UK) was used with diaminobenzidine as chromagen, as previously detailed,^29^,^30^ was used. For negative control, the primary antibody was substituted with antibody diluent. Normal breast tissue within sections was used as the internal positive control.

### Scoring

Mib-1 and PPH3 immunoreactivity in NCB and SES was scored (T.C.K.) in a blinded manner using a semiquantitative scoring system as previously published.^31^,^32^ The Mib-1 and PPH3 scoring system was different as both antibodies stained nuclei at different cell cycle phases. However, for both systems, only invasive tumour was scored, avoiding inflamed or necrotic regions.

#### Mib-1 scoring

At ×10 objectives, the area of greatest density of Mib-1 stained cancer cells was identified. Using a 10 × 10 eye-piece graticule, 1000 invasive breast cancer cells were counted using HPF (×400). Consecutive fields were used if >1 HPF was needed to count 1000 tumour cells. The Mib-1 growth fraction was calculated as the percentage of the 1000 breast cancer cells that were stained by Mib-1 irrespective of the intensity of immunoreactivity.^31^

#### PPH3 scoring

**Figure 1:**
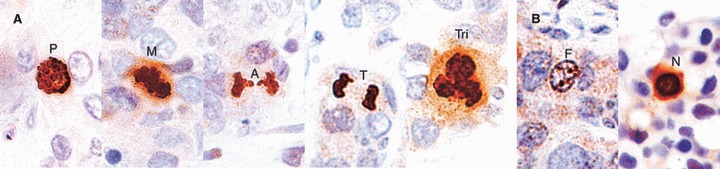
Photomicrographs showing PPH3 stained bona fide (**A**) and non-mitotic figures (**B**) (P, Prophase; M, Metaphase; A, Anaphase; T, Telophase; Tri, Tripolar mitotic figure; F, fine granular staining; N, intact nuclear membrane).

Sections were screened to identify the area of greatest mitotic activity using ×10 objectives. The number of PPH3-stained mitotic figures was counted in 10 consecutive HPFs (×400).^26^,^27^,^32^ Mitotic figures (MFs) included in the scoring were those with morphological features resembling normal mitosis phases (i.e. prophase, metaphase, anaphase and telophase) and abnormal mitoses, such as tripolar mitotic figures. PPH3-stained nuclei with fine granular staining or intact nuclear membrane were excluded as they are not in mitosis (Figure 1).

### Statistical analysis

Statistical analysis was performed using SPSS software version 15.0 for Windows (SPSS Inc., Chicago, IL, USA). Bland and Altman analysis^33^ was used to quantify numerically the mean difference and spread of the intra-observer reproducibility of the Mib-1 and PPH3 scoring methods. For each of the three proliferation measures (H&E, Mib-1 and PPH3), Bland and Altman analysis and Wilcoxon signed rank test (non-parametric *t*-test) were used to reveal any differences between the NCB scores and SES scores.

Two receiver–operating characteristic (ROC) analyses were used to assess the concordance of each of the three NCB proliferation measures with the gold standard. To achieve this, a non-parametric approach was used to compare the area under the two ROC curves (AUC)^34^ with a *P*-value of 0.05 used to denote statistical significance. The larger the AUC, the better the concordance with the gold standard. In the first ROC analysis, a cut-off of H&E mitotic score in SES (gold standard) of 1 was used (i.e. yes = grade 1; no = grade 2 and 3) to determine the NCB proliferation measure that best agreed with the gold standard in identifying low mitotic score breast cancers. The second ROC analysis used the cut-off of H&E mitotic score in SES of grade 3 (i.e. yes = grade 3; no = grade 1 and 2) to determine the NCB proliferation marker that best identified high-grade breast cancers.

## Results

### H&E mitotic counts

The H&E mitotic scores in the NCB and SES agreed in 64 of 101 tumours (63%; κ-value = 0.25) (Table 1). The corresponding levels of agreement for tubule formation, nuclear pleomorphism and histological grade were 78, 79 and 65% with κ-values of 0.52, 0.59 and 0.47, respectively.

**Table 1:** Comparison between mitotic score on needle core biopsy and surgical excision samples of breast cancers

	Mitotic frequency score on SES		
	
Mitotic frequency score on NCB	Score 1	Score 2	Score 3
Score 1	58	9	15

Score 2	1	3	11

Score 3	0	0	4

### Immunohistochemical proliferation markers

**Figure 2:**
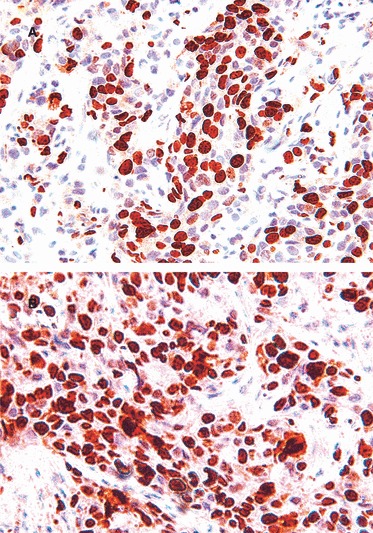
Photomicrographs showing a wide variation in intensity of Mib-1 nuclear staining pattern in a case with moderate (**A**) and high (**B**) Mib-1 growth fraction.

Mib-1 immunoreactivity was confined to the nucleus with varying intensity of reactivity (Figure 2) and high expression at the invasive tumour periphery. PPH3 staining was confined to the nucleus with strong, discrete, contrast-rich brown immunoreactivity of chromatin clumps (Figure 1).

**Figure 3:**
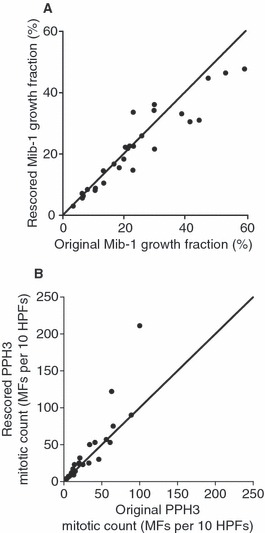
Scatter plots with line of equality showing intra-observer reproducibility of Mib-1 (**A**) and PPH3 (**B**) scoring. (MF, Mitotic figures; HPF, high power field).

Adequate intra-observer reproducibility was found in scoring both Mib-1 (mean difference of Mib-1 growth fraction of 2.1%; 95% limits of agreement between −8.4% and 12.6%) (Figure 3A) and PPH3 (mean difference of PPH3 mitotic count of 8 MFs; 95% limits of agreement between −40.5 MFs and 56.5 MFs) (Figure 3B).

### Comparison between proliferation measures

**Figure 4:**
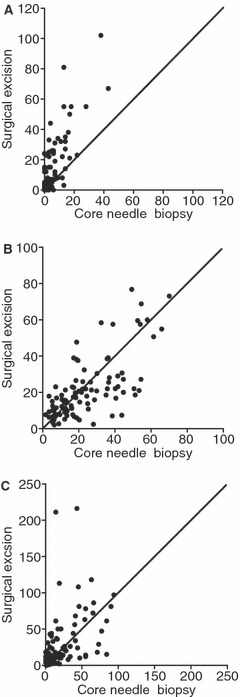
Scatter plots with line of equality showing H&E mitotic count (**A**), Mib-1 growth fraction (**B**) and PPH3 mitotic count (**C**) in needle core biopsy and surgical excision specimens for the 101 cases in the study.

Wilcoxon signed rank test found H&E mitotic count in NCB to be (range 0–43 MFs/10 HPF, mean = 5 MFs, median = 3 MFs) significantly lower (z = −6.18, *P* < 0.001, *n* = 101) than in SES (range 0–102 MFs/10 HPF, mean = 14 MFs, median = 5 MFs). A ratio of mean difference of 2.8 (95% limits of agreement between −4.3 and 10.0) was found between H&E mitotic count in NCB and SES (Figure 4A). However, on Wilcoxon signed rank test no significant difference was found (z = −1.50, *P* = 0.13, *n* = 101) between Mib-1 in NCB (range 1.6–70.1%, mean = 23.6%, median = 18.4%) and Mib-1 in SES (range 2.0–76.8%, mean = 21.2%, median = 17.1%) with a ratio of mean difference of 1.1 (95% limits of agreement between −0.5 and 2.7) (Figure 4B). No significant difference was found either (z = −1.79, *P* = 0.073, *n* = 101) between PPH3 in NCB (range 0–94 MFs/10 HPF, mean = 22 MFs, median = 13 MFs) and PPH3 in SES (range 0–216 MFs/10 HPF, mean = 29 MFs, median = 13 MFs) with a ratio of mean difference of 1.9 (95% limits of agreement between −2.7 and 6.6) (Figure 4C).

**Figure 5:**
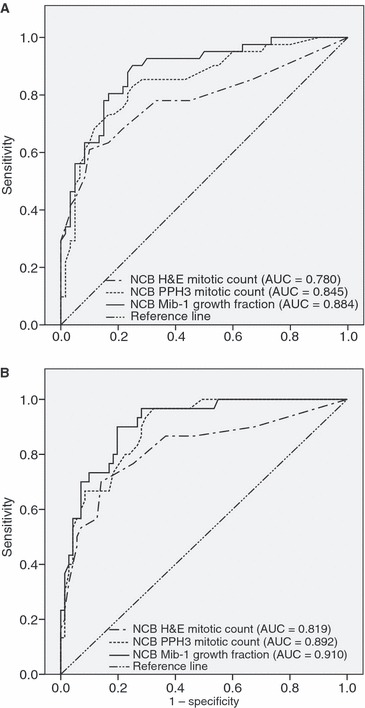
ROC analysis assessing concordance between NCB proliferation measures (H&E, Mib-1 and PPH3) with the gold standard of H&E mitosis grade in surgical excision specimens (SES) using a cut-off of H&E mitosis grade in SES of 1 (**A**) and 3 (**B**).

In the first ROC analysis (cut-off of H&E mitotic grade 1 in SES) (Figure 5A), the AUC for both Mib-1 (AUC = 0.884 ± 0.034) and PPH3 (AUC = 0.845 ± 0.041) in NCB was larger than AUC for conventional H&E mitotic count (AUC = 0.780 ± 0.051). However, only the AUC comparison between Mib-1 and H&E achieved statistical significance (*P* = 0.019), unlike the AUC comparison between PPH3 and H&E (*P* = 0.114).

In the second ROC analysis (cut-off of H&E mitotic grade 3 in SES) (Figure 5B), a similar situation was found. AUC for Mib-1 (AUC = 0.910 ± 0.029) and PPH3 (AUC = 0.892 ± 0.031) was larger than AUC for H&E (AUC = 0.819 ± 0.051). Only the comparison of AUC between Mib-1 and H&E achieved statistical significance (*P* = 0.019), unlike the AUC comparison between PPH3 and H&E (*P* = 0.091).

## Discussion

Proliferation is one of the strongest prognostic factors in breast cancer.^26^,^35^,^36^ Despite the various proliferation measures, routine H&E mitotic count is still preferred, as it is cheap, quick and does not require special equipment.^35^,^37^ However, underestimation of H&E mitotic count in NCB potentially affects clinical decisions. This pilot study was performed to investigate the possibility of using immunohistochemistry to improve NCB proliferation assessment.

### Reason for NCB underestimation of H&E mitotic count

Due to breast cancer heterogeneity in regard to proliferation,^38^ the inherent undersampling of the NCB procedure is unsurprisingly the most common cited reason for the underestimation.^7^–^9^ Although it may play a role, as 14 cases were excluded in this study due to insufficient NCB tumour material for microscopic assessment, it is unlikely to be the sole cause. Moreover, it is inconclusive as to whether increasing the amount of tissue retrieved during NCB will improve mitotic count agreement between NCB and SES.^1^,^39^–^42^

It was anticipated that tissue fixation could be a factor in discrepancies between NCB and SES mitotic counts. Fixation is usually rapid and uniform in NCB but may not be so in SES.^14^,^21^,^43^ Previous studies, including our own observation on effect of delay of fixation (unpublished data),^44^–^48^ have shown that there is a reduction in visibility of mitoses with increasing length of fixation delay. The adequacy of our fixation procedures is supported by the findings of our previously published study,^49^ which demonstrated that, using the same fixation procedures, excellent agreement was found between oestrogen receptor expression in SES and NCB, which is also sensitive to fixation delay. The limited effect of fixation time difference between NCB and SES in our study samples is also supported by our findings that mitoses are higher in SES than in NCB, contrary to what might be expected if slow fixation in SES resulted in loss of mitotic figures. Thus, it is unlikely that the difference in mitotic counts between SES and NCB is related to fixation time or time between surgery and fixation.

In this study, H&E mitotic count was significantly underestimated in NCB, as previously reported.^2^,^6^–^13^ However, NCB Mib-1 and PPH3 scores did not differ significantly from their respective SES scores.

Thus, a possible contributory factor to NCB underestimation of mitotic count may be that fragile mitotic figures in routine H&E slides might be obscured by crushing or traumatic damage of cancer cells during the NCB procedure^14^,^42^ that affects the visibility of mitoses in NCB rather than actual change in the number of dividing cells.

### Immunohistochemical proliferationmarker in NCB

#### Mib-1

The distribution of Mib-1 growth fraction in this study was consistent with previous reports.^38^,^50^–^56^ Mib-1 in NCB specimens was better at identifying low and high mitotic counts in the surgical specimen than routine H&E mitotic count in NCB specimens (Figure 5).

Patients with high Mib-1 growth fraction are found to be good candidates for neoadjuvant chemotherapy because highly proliferating cells are responsive to antimitotic drugs.^22^ As fragile mitotic cells might be damaged during the NCB procedure,^14^ Mib-1 scores may be less likely to be underestimated in NCB specimens due to Mib-1 labelling of G_1_, S and G_2_ phases besides M phase, unlike H&E and PPH3. However, this labelling might undermine its prognostic value as cells may have undergone apoptosis before entering M phase.^21^,^22^

#### PPH3

The PPH3 pattern of nuclear immunoreactivity in this study was comparable to previous studies.^24^,^26^,^27^ Although not achieving statistical significance, PPH3 on NCB was found to be better at identifying both low and high mitotic grade breast cancer than routine H&E.

The distinct brown PPH3 immunoreactivity enabled easy and rapid screening for high proliferation even using low objectives. PPH3 staining could identify prophase nuclei easily and distinguish mitotic figures from unstained apoptotic or necrotic nuclei,^26^ unlike H&E.^20^ These are crucial in NCB, where specimens might be damaged during the NCB procedure, obscuring the identification of mitotic figures.

## Conclusion

This study has confirmed previous studies showing that mitotic count assessed on H&E sections of NCB underestimates the H&E mitotic count in SES. The immunohistochemical markers Mib-1 and PPH3 in NCB showed a better concordance with the H&E mitotic count in SES, but further prospective studies are needed to provide standardization of scoring methodology^57^ and cut-offs.^25^–^27^,^52^,^58^,^59^

## Abbreviations

AUC: area under the curve
FFPE: formalin-fixed paraffin-embedded
H&E: haematoxylin and eosin
HPF: high-power field
MF: mitotic figure
NCB: needle core biopsy
PPH3: phosphorylated histone H3
ROC: receiver–operating characteristic
SES: surgical excision specimen(s)
